# From technological closed-loop to human-machine trust: a systematic review of ethical and communication challenges of brain-computer interface in elderly care

**DOI:** 10.3389/fdgth.2026.1877688

**Published:** 2026-08-28

**Authors:** Kun Fu, Peize Li, Xinxi Peng, Shuangcheng Li, Yifan Zhao, Xi Chen, Yi Ding, Chenxi Ye

**Affiliations:** School of Culture and Creativity, Beijing Normal-Hong Kong Baptist University, Zhuhai, China

**Keywords:** ageing, brain-computer interface, communication challenges, digital health ethics, elderly care, ethics, human-machine interaction, neuroethics

## Abstract

As the global population ages, brain–computer interfaces (BCIs) have emerged as a promising technological pathway for restoring communication, motor function, and cognitive support in elderly care settings. However, the ethical and communication challenges accompanying BCI deployment in these contexts remain insufficiently understood. This systematic review identified 177 studies meeting the inclusion criteria from 12,423 records. Through comprehensive analysis, six core ethical themes were identified: privacy and data security, informed consent and autonomy, personhood and identity, technical risks and safety, equity and accessibility, and risk-benefit trade-offs. Beyond cataloging these ethical challenges, this review proposes a three-level analytical framework encompassing human-computer interaction, interpersonal communication, and public opinion dissemination to examine how ethical risks manifest as communication breakdowns across the closed-loop BCI process of neural signal acquisition, intention decoding, and external feedback. Furthermore, a three-dimensional mapping integrating ethical issues, communication challenges, and application scenarios (rehabilitation assistance, communication assistance, monitoring and prevention, and emotional support) is constructed to reveal scenario-specific ethical configurations. The findings indicate that BCI's ethical risks are not peripheral but structurally embedded in the technology's interaction logic-decoding uncertainty can translate into care misjudgment, neural data inferability extends privacy risks beyond information leakage to psychological exposure and power asymmetry, and technology-mediated communication creates structural tensions in trust and responsibility. The review concludes that responsible BCI deployment in elderly care requires not merely algorithmic improvement but the systematic engineering of ethical principles into care processes, including uncertainty disclosure at the interface level, data minimization and layered informed consent at the institutional level, and interdisciplinary collaboration with long-term support systems.

## 1 Introduction

Ageing is accompanied by well-documented declines across multiple cognitive domains, i.e., processing speed, working memory, selective attention, and new learning, as well as in sensorimotor function, which is driven by prefrontal atrophy, reduced dopamine modulation, and white matter deterioration (1, 2). Even cognitively healthy older adults show measurable deficits that compromise quality of life and limit social participation (3, 4). The most acute expression of these trajectories is dementia: global prevalence rose by over 160% between 1990 and 2019 and is projected to affect 152 million individuals by 2,050 (5), at a cost of 9.4% of total global health spending, with informal caregiving already accounting for more than half of this total (6). Prior to dementia, sensorimotor deterioration produces a concrete mortality risk, with 28%–35% of adults aged 65 and above suffering from falling or tripping annually, which is the second leading cause of unintentional injury-related death worldwide (7, 8). Social isolation further accelerates decline: a meta-analysis of 86 prospective cohort studies (7).quantifies social isolation as independently associated with a 35% increase in all-cause mortality. These converging pressures are intensified by the fundamental inadequacy of pharmacological responses: no available drug halts age-related neuronal loss, and even the latest anti-amyloid therapies slow decline without reversal, carry serious cerebrovascular risks, and cost approximately US$28,000 per patient annually (9).

The clinical justification for neurotechnological intervention based on Brain-Computer Interface (BCI) therefore rests on the structural inadequacy of all aforementioned alternatives. By definition, a BCI acquires neural signals (via non-invasive scalp electrodes, cortical surface arrays, or intracortical implants), extracts meaningful features, and translates them in real time into commands that control external devices, thereby bypassing peripheral neuromuscular pathways progressively compromised by neurological disease and normal ageing (10). Reviews of EEG-based neurofeedback and BCI interventions report promising domain-specific gains in older adults, notably in attention and working memory, with a recent meta-analysis identifying moderate effect sizes for working memory improvement (11).

Central to this review's contribution is a clear specification of what “communication” refers to in the BCI context. We distinguish three distinct, non-interchangeable registers, each carrying its own ethical implications and each to be applied systematically in Sections 5, 6, and 7:

(i) Functional communication restoration: the use of BCI to restore or augment an individual's capacity to convey intentions, commands, or speech to the external environment (e.g., speech neuroprosthetics, P300-based character selection, motor command decoding for populations with ALS, locked-in syndrome, or severe motor impairment).
(ii) Interpersonal care communication: the exchange of information, meaning, and trust between the BCI user and their caregivers, clinicians, and families, encompassing informed consent, technical explanation, expectation management, and care decision-making.
(iii) Public and institutional communication: the broader discursive field in which BCI technology is represented, evaluated, and governed, including media coverage, professional discourse, and regulatory deliberation.

These three levels are distinct and operate on different levels. For example, a failure in signal decoding (functional level) does not operate identically to a breakdown in clinician-patient explanation (interpersonal level), nor to distorted public expectations shaped by media exaggeration (institutional level). The three-level framework thus organises the communication challenge analysis and enables precise mapping of where and how ethical risks manifest as communication breakdowns.

Over the last decade, BCI research has diversified from controlled laboratory paradigms toward clinically relevant and consumer-oriented applications for elderly care (12). Invasive systems have demonstrated technical feasibility of motor function restoration (13, 14), and speech neuro-prosthetics have achieved near-conversational decoding speeds (15, 16)(Willett et al., 2023; Metzger et al., 2023). Non-invasively, P300-based spellers and EEG-based neurofeedback have shown gains in memory, attention, and executive function in older adults and MCI patients (17–19), though inter-study variability in protocol design still limits synthesis.

These technical advances described above thus produce a structural duality. On one hand, BCI offers genuine promise for public health: restoring communicative and motor agency can restore independence and improve psycho-social wellbeing in populations where these capacities are progressively eroded (20, 21). On the other hand, the same capabilities introduce a qualitatively novel category of ethical and privacy risks that existing biomedical or data protection frameworks cannot adequately manage (22). articulate this distinctiveness: deep-learning systems applied to neural recordings can reliably establish statistical correlations between EEG signals and specific cognitive, emotional, and conative states, thereby creating unprecedented infrastructure for involuntary mental surveillance. Neural signals, once decoded, can then be re-synthesised into personally identifying outputs (voice models, emotional profiles) that retain inferential sensitivity across technical, organisational, and legal boundaries; a tiered legal analysis places brainwave patterns in the highest protection tier, equivalent to biometric fingerprints (23). Szoszkiewicz and Yuste (24) further note that neural data disclose our most intimate cognitive processes and can forecast future behaviour, health risks, and cognitive performance, which is qualitatively different from conventional health data. These inferences extend to mood states, attentional patterns, and susceptibility markers highly consequential in insurance, employment, and caregiving.

The scale and novelty of these risks have begun to attract institutional responses, though the regulatory landscape remains fragmented. Evidence from the digital mourning context, a neighboring domain, offers a useful parallel. Fu et al. (25) found that ethical concern exerted a significant negative effect on behavioural intention, indicating that heightened ethical awareness directly reduces willingness to adopt AI-mediated remembrance technologies. Conversely, grief perception positively influenced both behavioural intention and actual use behaviour, suggesting that emotional vulnerability can paradoxically drive technology acceptance in ethically charged domains (25). These findings illustrate the dual tension—ethical apprehension against emotional need—that also characterises the BCI landscape for elderly users. A review of BCI device privacy policies Yang & Jiang (26) found that most manufacturers do not technically guarantee inaccessibility of neural data to their own personnel, and General Data Protection Regulation (GDPR) as well as other analogous frameworks lack the specificity to govern BCI-derived inferences. Scholars have proposed multi-pillar governance frameworks combining neural data legislation, ethical guidelines, and codification of neuro-rights (26); Gordon and Seth (27) argue that “cognitive liberty”, which is the right to neural self-determination, must be enshrined before clinical deployment scales. The European Parliament's STOA panel (28) concluded that human rights instruments alone are insufficient, and data law reform must proceed in parallel with neuro-rights codification.

What makes this ethical scrutiny distinctly urgent for older adults is not merely their heightened clinical vulnerability, but the structural mismatch between what BCI systems demand and what this population can reliably provide: stable attentional resources, consistent neural signal characteristics, and the cognitive capacity to navigate complex consent processes. This mismatch transforms otherwise general ethical concerns, such as privacy, autonomy, safety, into acute, and operationally specific challenges. A technology that decodes neural signals probabilistically, deployed in a care setting where signal quality degrades with age and disease progression, and governed by consent frameworks designed for cognitively intact adults, embeds those questions directly into the moment-to-moment practice of care.

To address this gap, the present systematic review advances beyond prior literature in two ways. First, we provide a comprehensive synthesis of ethical challenges across six core themes: (1) *privacy and data security*, (2) *informed consent and autonomy*, (3) *personhood and identity*, (4) *technical risks and safety*, (5) *equity and accessibility*, and (6) *risk-benefit trade-offs*. These themes are derived from a systematic analysis of 186 eligible studies (from 12,423 initial records) and are defined operationally in Section 3. Second, we propose an analytical framework that maps how ethical risks manifest as communication breakdowns across the three levels of BCI-mediated interaction defined above. Furthermore, we construct a three-dimensional mapping (Section 7) that integrates ethical issues, communication challenges, and application scenarios (rehabilitation assistance, communication assistance, monitoring and prevention, emotional support), revealing scenario-specific ethical configurations. This framework demonstrates that BCI's ethical risks are structurally embedded in the technology's interaction logic: decoding uncertainty translates into care misjudgment, neural data inferability extends privacy risks beyond information leakage to psychological exposure and power asymmetry, and technology-mediated communication creates structural tensions in trust and responsibility. The review concludes that responsible BCI deployment in elderly care requires the systematic engineering of ethical principles into care processes, including uncertainty disclosure at the interface level, data minimisation and layered informed consent at the institutional level, and interdisciplinary collaboration with long-term support systems.

## 2 Methods

This systematic review was prospectively submitted to the PROSPERO international register of systematic reviews on 17 June 2026, with an active registration record pending official audit. Owing to an oversight of the journal's pre-registration specification at the initial research stage, the registration was completed following preliminary literature deduplication and title/abstract screening, rather than prior to all research activities. Nevertheless, all core prespecified protocols documented in the PROSPERO form — including database search strategies, triple inclusion/exclusion criteria, deductive thematic analysis rules, methodological quality appraisal standards and the construction logic of the three-dimensional analytical framework — were finalized before full-text data extraction and qualitative synthesis. No adjustments were made to the research scheme after reviewing eligible literature, and all original screening and coding records are retained to ensure full reproducibility.

**Figure 1:**
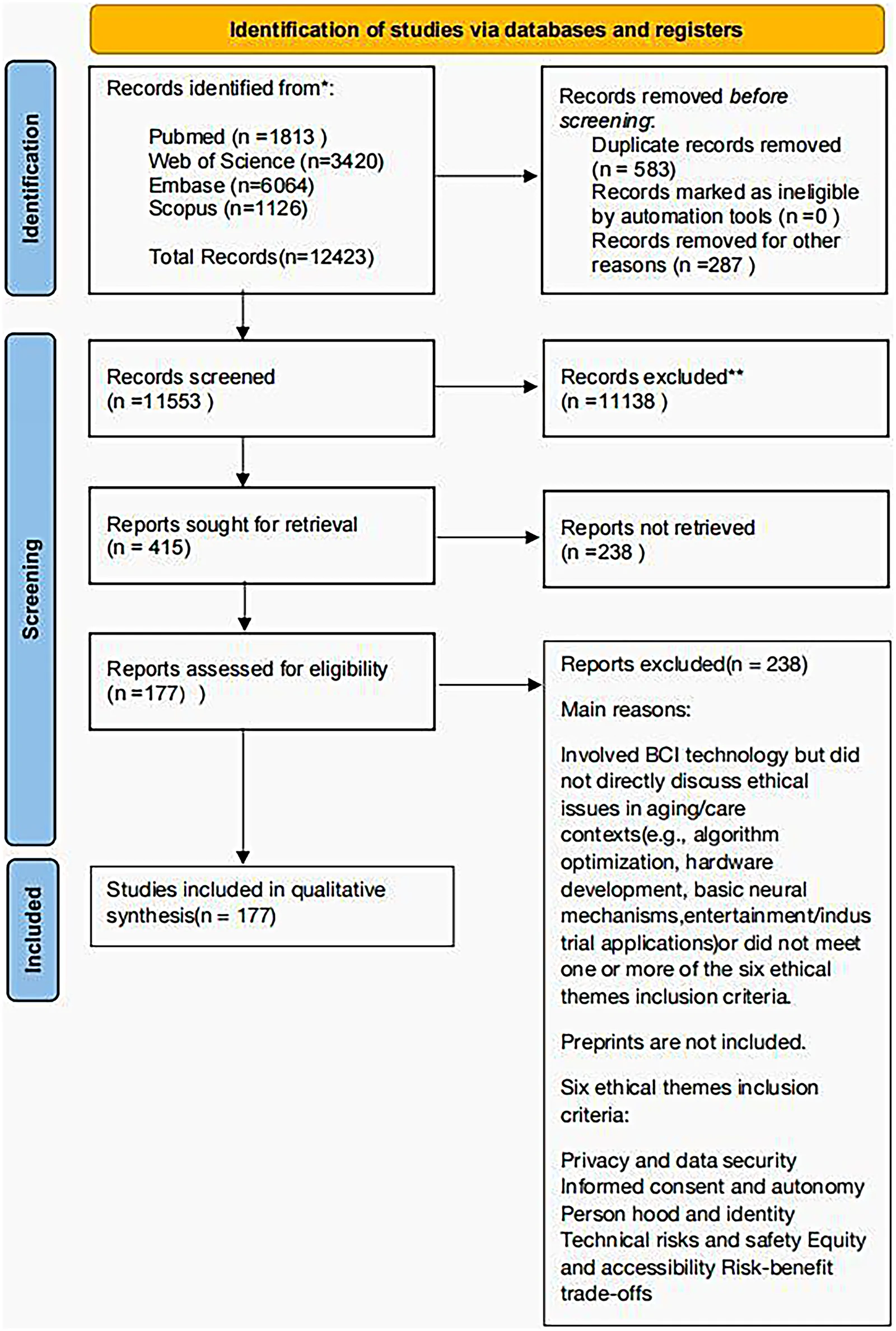
PRISMA-style flowchart illustrating the selection process for studies via databases and registers, including records identified, screened, excluded, and the main reasons for exclusion based on six ethical themes such as privacy, consent, and equity.

The PRISMA 2020 Flow Diagram for Systematic Reviews is shown in Figure 1.

This study's literature identification and screening process strictly follows standard reporting guidelines in the fields of systematic reviews ，including the PRISMA guidelines. This framework provides standardized procedures and transparent reporting structures for systematic reviews. Figure 1 illustrates the screening process for this study.

Systematic searching was completed on February 28, 2026, covering four major databases: PubMed, Web of Science, Embase, and Scopus. The search scope was limited to title, abstract, and keyword fields. The search strategy was constructed around core themes including “brain–computer interface,” “aging/elderly care,” and “ethics/safety.” The specific search strategy was as follows:

(“brain-computer interface” OR BCI OR “brain machine interface” OR “neural interface” OR “neuroprosthetic” OR “cognitive prosthesis” OR EEG OR “electroencephalography” OR “neurotechnology”) AND (elder* OR aging OR ageing OR geriatric* OR “old* adult” OR “older people” OR “senior” OR “age-related” OR “long-term care” OR “nursing home” OR “assisted living” OR “Alzheimer” OR dementia OR “cognitive decline” OR “neurodegenerative disease” OR “Parkinson” OR “stroke rehabilitation”) AND (ethic OR “ethical issue*” OR “responsible research and innovation” OR RRI OR fairness OR bias OR “algorithmic bias” OR equity OR autonomy OR “agency” OR “informed consent” OR “decision making capacity” OR privacy OR “data protection” OR “security” OR justice OR accessibility OR “digital divide” OR “social impact” OR “human dignity” OR “personhood” OR “welfare” OR “beneficence” OR “clinical translation” OR “patient acceptance” OR “attitude” OR “trust” OR “stigma”)

During the identification phase, 12,423 literature records were obtained. After automatic deduplication using Zotero (v8.0.3) removed 583 duplicate records, manual review further removed 287 additional records (including duplicates between preprint and formal publication versions, cross-database duplicates, etc.), resulting in 11,553 unique records entering the screening process.

### 2.1 Screening process

The literature screening in this review was conducted in two phases—title/abstract screening and full-text screening—with a three-tier quality assurance mechanism consisting of “execution,” “content verification,” and “process audit.” The specific division of labor is specified as follows:

(1) Screening execution. Title/abstract screening and full-text screening were performed by the first reviewer (P.L.). All screening decisions were based on predefined inclusion criteria (the triple requirement of “BCI technology+aging/care scenarios+ethical issues”) and an exclusion reason classification system (see below).

During the title and abstract screening phase, studies were primarily screened based on the triple inclusion criteria of “BCI technology+aging/care scenario+ethical issues.” Excluded studies mainly fell into three categories:

(a) Irrelevant topic studies (primary exclusion source): A large volume of literature involves EEG, neuroimaging, or brain-computer interface-related technologies but does not simultaneously meet ethical or care scenario requirements. Specifically included: purely technical/engineering studies (such as algorithm optimization, signal processing, and model training); non-medical or non-care application scenarios (such as entertainment, gaming, or industrial control); basic neuroscience research (not involving practical application contexts); and experimental studies that do not discuss any ethical issues.
(b) Terminology ambiguity issues: In some studies, “BCI” is used to refer to other medical or technical concepts, such as bilateral cochlear implantation, behavioral change interventions, etc., which do not align with the research topic of this study.
(c) Non-compliant technical paradigms: Some studies involve brain signal acquisition (such as EEG, MEG) but do not form closed-loop brain-computer interaction systems (i.e., lacking the “brain signal-system feedback-user regulation” interaction mechanism), thus not meeting the operational definition of brain-computer interfaces.

The majority of excluded records failed to simultaneously satisfy the triple inclusion criteria of “BCI + aging/care scenario + ethical issues,”, addressing instead only single or partial dimensions in isolation. To avoid omitting potentially relevant studies, for literature that did not explicitly present ethical dimensions in titles and abstracts but was highly relevant to the research topic, researchers adopted a prudent retention strategy during screening and further confirmed inclusion eligibility during full-text assessment.

This phase excluded 11,138 records, ultimately retaining 415 articles for full-text assessment.

During the full-text assessment phase, screening was conducted according to six major ethical theme inclusion criteria, including: privacy and data security, informed consent and autonomy, personhood and identity, technical risks and safety, equity and accessibility, and risk-benefit trade-offs. Ultimately, 177 articles met inclusion criteria and entered qualitative synthesis analysis.

During eligibility assessment, 238 studies were excluded, primarily because although they involved BCI technology, they did not directly discuss ethical issues in care or aging contexts. These studies mainly focused on signal processing algorithm optimization, hardware development, basic neural mechanisms, or entertainment/industrial applications. Simultaneously, we assessed ethical issues involved in clinical and preclinical experiments, which contributes to subsequent consideration of ethical issues. Table 1 shows them.

(1) Content verification of included literature. All 177 finally included articles were independently content-verified by the second reviewer (X.P.), with a focus on checking: (a) whether each article simultaneously satisfied the three inclusion requirements; (b) whether the classification into the six ethical themes was accurate and appropriate; and (c) whether the key data extraction items (study type, application scenario, main ethical findings, etc.) were complete and correct. Any disagreements identified during the verification process were resolved through discussion and consultation between the two reviewers; if consensus could not be reached, the third reviewer (K.F.) or (C.Y.) made the final decision.
(2) Process audit of screening procedures. To ensure the overall standardization and traceability of the screening process, the third reviewer (K.F.) conducted an independent audit of the entire screening procedure, including: spot-checking exclusion records from the title/abstract phase to confirm the appropriateness of exclusion reason code applications; and checking the logical consistency of literature counts across phases in the PRISMA flow diagram. Any anomalies or deviations identified during the audit were returned to the screening execution stage for correction and confirmed through team discussion.
(3) Exclusion reason classification system. Prior to the formal initiation of screening, the research team, based on preliminary literature scoping, pre-established three main categories of exclusion reason codes: (a) “Theme irrelevance"—involving EEG, neuroimaging, or BCI-related technologies but not simultaneously satisfying the ethical or care scenario requirements; (b) “Terminological ambiguity"—where “BCI” in the text refers to other technical concepts not intended in this study (e.g., bilateral cochlear implants, behavioral change interventions, etc.); (c) “Technical paradigm mismatch"—involving brain signal acquisition but without constituting a closed-loop brain–computer interface system. In the full-text screening phase, the above codes were further refined into inclusion/exclusion comparison criteria corresponding to the six ethical themes. Through the above three-tier “execution–content verification–process audit” mechanism and full decision documentation, the accuracy and logical traceability of the entire screening process were systematically ensured.

**Table 1:** Technical stages, study types, applications, sample characteristics, and major ethical risks in BCI research.

Technology Stage	Research Type	Typical Applications	Sample Characteristics	Major Ethical Risks	Ethical Research Focus
Preclinical	Basic experimental research, animal experiments, *in vitro* cell experiments	R&D of core BCI technologies, optimization of neural decoding algorithms, hardware testing of implantable devices	Laboratory animals (rodents, non-human primates), *in vitro* cultured neural cells/organoids, no human subjects	1. Lack of laboratory animal welfare and ethical review; 2. Ethical controversies over non-human primate experiments; 3. Insufficient prediction of the effectiveness and safety of translating experimental results to humans; 4. Scientific research data fraud and selective publication of results	1. Implementation of laboratory animal ethical norms; 2. Registration and transparency of preclinical research; 3. Repeatability verification of animal experiment results; 4. Risk prediction and pre-ethical review before clinical translation
Human Experimental	Human experiments on healthy subjects, First-in-Human (FIH) trials, feasibility studies	Human safety verification of BCI devices, neural decoding accuracy testing, user experience optimization	Healthy adult volunteers, a small number of subjects with mild dysfunction, usually small sample size (*n* < 30), no long-term implantation	1. Imbalanced risk-benefit ratio for healthy subjects; 2. Inadequate informed consent, insufficient awareness of potential risks among subjects; 3. Accidental neurological damage during the experiment; 4. Lack of privacy protection for experimental data; 5. Result exaggeration and publication bias	1. Ethical review standards for experiments on healthy subjects; 2. Design of hierarchical informed consent process; 3. Real-time safety monitoring during the experiment; 4. Anonymization and privacy protection of human experiment data; 5. Publication norms for negative results
Clinical Trial	Phase I/II/III clinical trials, multicenter clinical studies, Randomized Controlled Trials (RCT)	Clinical effectiveness verification, indication expansion, and long-term safety evaluation of BCI devices	Patients with target diseases (e.g., spinal cord injury, amyotrophic lateral sclerosis, severe paralysis), gradually expanding sample size, multicenter coverage, with clear inclusion and exclusion criteria	1. Insufficient protection of autonomous decision-making ability of vulnerable groups (patients with severe disability, cognitive impairment); 2. Lack of registration and transparency of clinical trials; 3. Non-standard reporting and handling of adverse events; 4. Vulnerabilities in privacy and security management of clinical data; 5. Selective publication of trial results and conflicts of interest	1. Surrogate decision-making and informed consent norms for vulnerable groups; 2. Full-process registration and result disclosure of clinical trials; 3. Standardized reporting and ethical review of adverse events; 4. Full-life cycle safety management of clinical data; 5. Disclosure and management of conflicts of interest in clinical trials
Long-term Invasive Implant	Post-marketing clinical follow-up, real-world studies, cohort studies of long-term implant users	Evaluation of long-term use effect, complication monitoring, and quality of life improvement evaluation of BCI devices	Long-term users who have completed surgical implantation of BCI devices, with a follow-up period usually ≥1 year, covering users of different ages and disease types	1. Chronic neurological damage and personality changes after long-term implantation; 2. Lack of long-term safety and effectiveness data of the device; 3. Insufficient protection of users' long-term autonomous decision-making ability and right to withdraw; 4. Risk of privacy leakage of long-term follow-up data; 5. Lack of emergency response mechanisms for device failures	1. Full-life cycle ethical management of long-term implant users; 2. Dynamic informed consent mechanism for long-term follow-up; 3. Long-term monitoring and ethical evaluation of neurological function and personality changes; 4. Secure storage and compliant use of long-term follow-up data; 5. Emergency response to device failures and protection of users' rights and interests

### 2.2 Quality assessment

Given the diversity of study types included in this review, a type-stratified quality assessment strategy was adopted. Quantitative studies (*n* = 98) were assessed using the JBI Checklist for Analytical Cross-Sectional Studies (8 items) (29); qualitative studies (*n* = 27) were assessed using the JBI Checklist for Qualitative Research (10 items) (30); review articles (*n* = 51) were assessed using AMSTAR 2 (31), with particular focus on the two critical domains of protocol preregistration and risk of bias assessment; the single mixed-methods study (*n* = 1) was assessed using both JBI quantitative and qualitative checklists, with the overall rating determined by the lower of the two scores (31).

All quality assessments were independently performed by the primary reviewer (P.L.) and subsequently verified by a second reviewer (K.F.). Disagreements were resolved through discussion, with a third reviewer consulted when necessary.

As shown in Table 2, among the 177 included studies, 54 (30.5%) were rated as high quality, 93 (52.5%) as moderate quality, and 30 (16.9%) as low quality. Studies rated as moderate or above comprised 83.1% of the total, confirming that the overall evidence base is robust to support the analytical framework developed in this review. Low-quality studies consisted primarily of early exploratory research or conference papers with incomplete methodological reporting; these were used only as supplementary references in the comprehensive analysis and framework construction.

**Table 2:** Quality assessment results of included studies (*n* = 177).

Quality Level	Number of Studies	Proportion
High Quality	54	30.5%
Moderate Quality	93	52.5%
Low Quality	30	16.9%
Total	177	100.0%

### 2.3 Data extraction and synthesis

The data extraction and synthesis process followed four steps:

- Step 1: Structured data extraction. For each included article, two reviewers independently extracted key information, including: first author and year, country/region, study type (quantitative/qualitative/review/theoretical), research objective, BCI technology type (invasive/non-invasive/hybrid), application scenario (rehabilitation/communication/monitoring/emotional support), primary ethical themes, key findings/perspectives, research methods and sample characteristics (if applicable), and ethical recommendations/conclusions. The extracted literature information was compiled into a coding table and managed using Microsoft Excel.
- Step 2: Initial coding and theme generation. The coding process adopted a combined “deductive and inductive” strategy.

Deductive level: Drawing on core topics from existing neuroethics and BCI ethics literature (32); Burwell et al.'s 2017 ethical theme classification (33), six preliminary coding categories were pre-established: privacy and data protection, informed consent and autonomy, personhood and identity, technical risks and safety, fairness and accessibility, and risk–benefit trade-offs. This pre-established framework provided a clear structural starting point for the coding work.

Inductive level: Building on the deductive framework, reviewers conducted open coding of the literature to identify emerging themes and sub-dimensions not fully covered by existing frameworks. For example, “trust formation in closed communication loops” and “ethical implications of decoding uncertainty” were new analytical dimensions induced and refined from the literature.

- Step 3: Theme integration and framework construction. After coding completion, we conducted a cross-relational analysis mapping the extracted ethical themes against the types of communication challenges. Specifically, we mapped each ethical theme (e.g., “privacy”) to different stages of the complete BCI communication closed loop (signal acquisition, intent decoding, external feedback), tracing the generation and transformation mechanisms of specific ethical issues within the communication chain. On this basis, we further conducted a stratified analysis of ethical–communication issues by application scenario (rehabilitation assistance, communication assistance, monitoring and prevention, emotional support), ultimately constructing a three-dimensional “ethical issues–communication challenges–application scenarios” mapping framework.
- Step 4: Assurance of analytical consistency. The two reviewers conducted independent coding tests on 177 articles. After the coding framework was clarified and calibrated, the two reviewers continued to independently code the remaining articles. Regular consistency checks were conducted during the coding process. In cases of coding disagreement (e.g., the attribution of ethical themes for a particular article, or whether a certain ethical issue should be classified under privacy or autonomy), resolutions were reached through discussion; if consensus could not be achieved, the third reviewer (K.F.) made the final decision.

**Figure 2:**
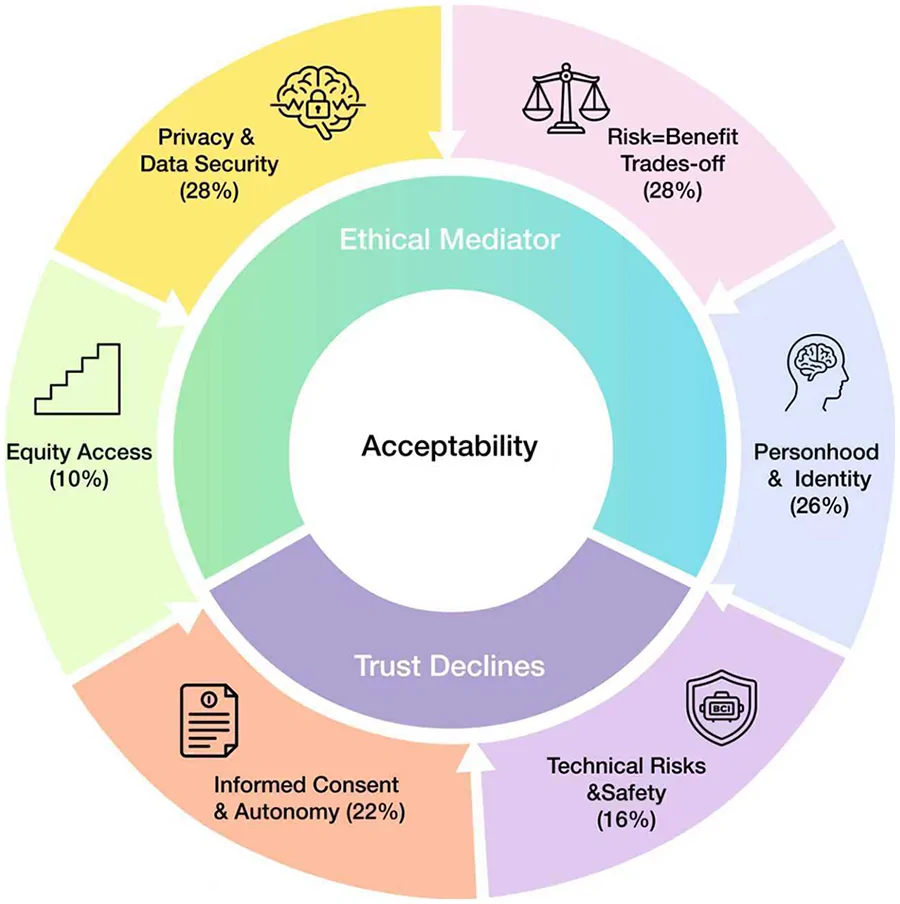
Circular diagram illustrating factors influencing acceptability, divided into six sections: Privacy and Data Security (twenty-eight percent), Risk-Benefit Trades-off (twenty-eight percent), Personhood and Identity (twenty-six percent), Technical Risks and Safety (sixteen percent), Informed Consent and Autonomy (twenty-two percent), Equity Access (ten percent). Central rings show the concepts Ethical Mediator and Trust Declines.

During the synthesis process, Microsoft Excel was used for coding management and categorical counting; no specialized qualitative data analysis software (e.g., NVivo) was employed. The classification of ethical themes is detailed in Table 2. Individual articles could involve multiple ethical themes, which was permitted in our coding system—each article could be simultaneously assigned to multiple thematic categories according to the relevant dimensions addressed in its content. For example, an article discussing both privacy and autonomy could be coded under both themes. Figure 2 (interrelationships among ethical challenges) was precisely drawn based on such cross-coded data, presenting the associative pathways between different ethical themes.

### 2.4 Study characteristics and comparison with existing reviews

**Figure 3:**
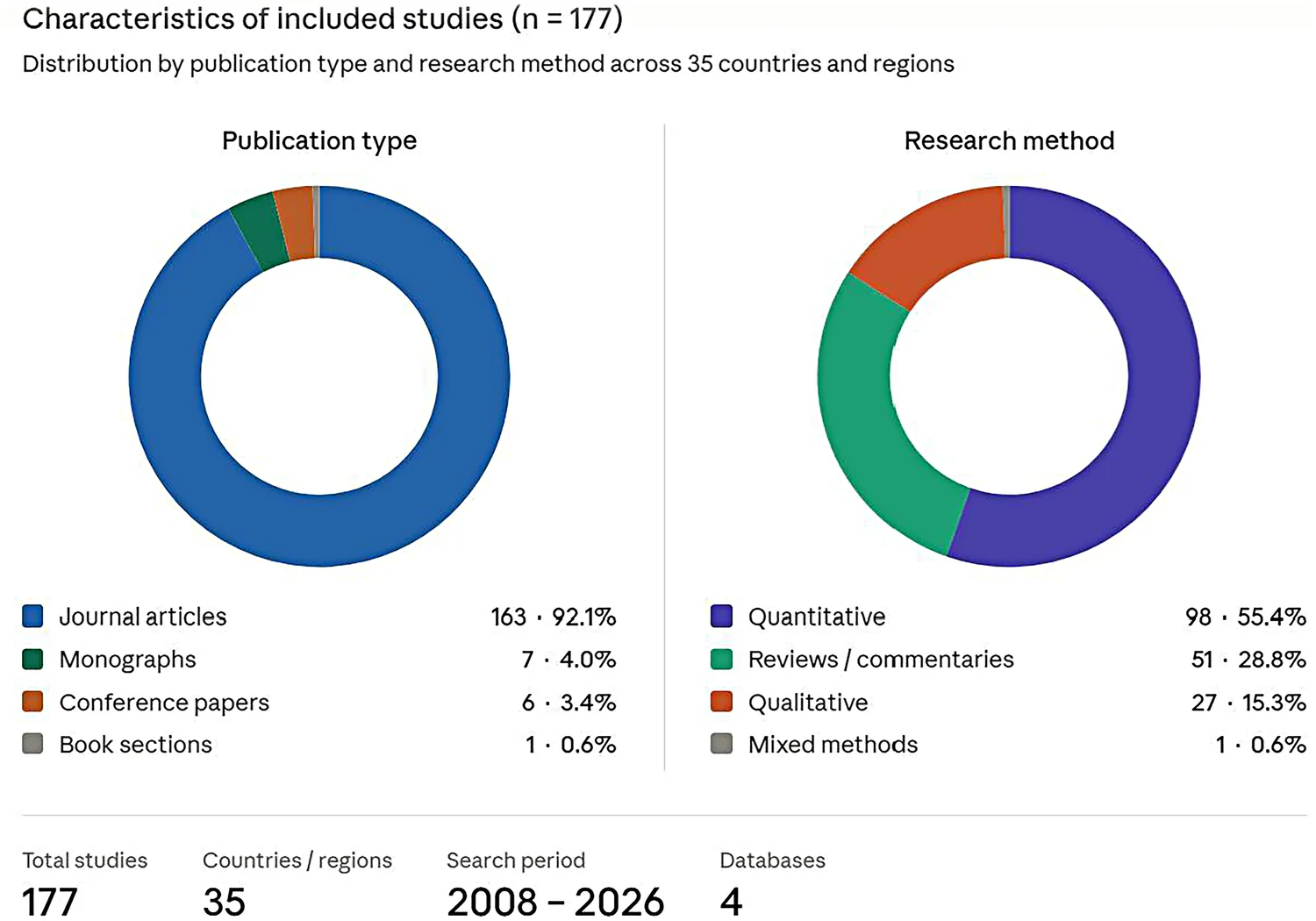
Two doughnut charts compare characteristics of 177 included studies. On the left, publication type shows 92.1 percent as journal articles, 4 percent monographs, 3.4 percent conference papers, and 0.6 percent book sections. On the right, research methods are 55.4 percent quantitative, 28.8 percent reviews or commentaries, 15.3 percent qualitative, and 0.6 percent mixed methods. The studies cover thirty-five countries or regions, span from 2008 to 2026, and use four databases.

The basic characteristics of the included studies are as follows: 163 journal articles (92.1%), 6 conference papers (3.4%), 7 book chapters/monographs (4.0%), and 1 book section (0.6%) (see Figure 3). In terms of research methods, the included studies present a diversified structure, comprising 98 quantitative studies (55.4%), 51 reviews (28.8%), 27 qualitative studies (15.3%), and 1 mixed-methods study (0.6%). Regarding geographical distribution, based on all authors' institutional affiliations, the studies span 35 countries and regions, including the United States, China, the United Kingdom, Canada, Germany, and others (see Figure 3).

In comparison with existing reviews, previous studies such as Andreas Kögel et al. (34) and Marcello Ienca and Roberto Andorno (32) primarily addressed brain-computer interface issues from neuroethical or technology governance perspectives, including neural privacy, autonomy, and legal frameworks. However, these studies mostly focused on single ethical dimensions or early developmental stages. In contrast, this study covers the research peak period after 2019 in terms of time span and systematically integrates six major ethical themes, further constructing a three-dimensional analytical framework of “ethical issues-communication challenges-application scenarios,” thereby more comprehensively addressing the complex ethical issues of brain-computer interfaces in aging applications.

In terms of methodological distribution, included studies show obvious diversification trends. Quantitative studies primarily employ questionnaires, system performance evaluations, and standardized scales (such as NASA-TLX, QUEST 2.0, Fugl-Meyer assessment); qualitative studies mainly use semi-structured interviews, focus groups, and ethnographic methods; mixed-methods studies combine both to enhance result interpretability. From a temporal trend perspective, related research grew significantly after 2019, particularly concentrating in BCI ethics, non-invasive technology, and rehabilitation application fields.

Acceptance studies of BCI technology in care scenarios indicate that potential users (especially ALS patients, spinal cord injury patient groups) have core demands for BCI centered on communication autonomy, device portability, long-term use safety, and functional accuracy. Most respondents list “command recognition accuracy ≥90%” as the primary expectation (35). Technical system-level research points out that EEG-based BCI faces technical bottlenecks such as insufficient signal stability, poor electrode adaptability, and operation fatigue. Most older users and disabled users report that non-invasive electrodes are prone to signal interruption due to scalp laxity, hair interference, and muscle tremors, and prolonged use easily produces cognitive fatigue. The aforementioned technical bottlenecks will be further analyzed in the dissemination mechanism and challenges section (34, 36, 37).

**Figure 4:**
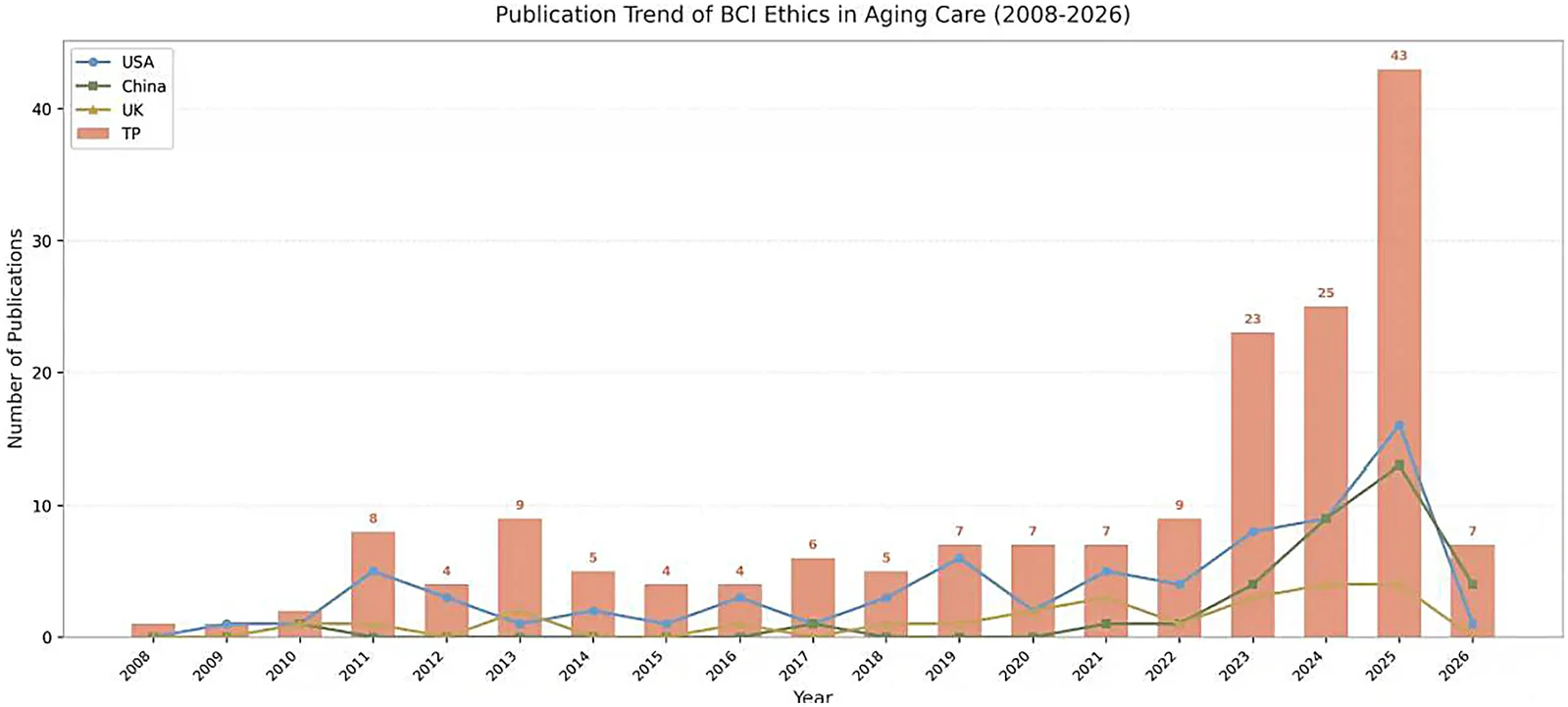
Line and bar chart showing the publication trend of BCI ethics in aging care from 2008 to 2026, with bars representing total publications and lines indicating publication numbers in the USA, China, and the UK. Significant increase is observed after 2022, peaking in 2025 at forty-three total publications, then declining.

Figure 4 shows the trend of publications by year.

## 3 Classification and comprehensive analysis of six major ethical dilemmas in BCI elderly care applications

Based on systematic analysis of the included literature, the ethical challenges of BCI in elderly care applications can be summarized into six core themes. The theoretical connotations and empirical foundations of each theme are elaborated below.

### 3.1 Privacy and data security ethics

#### 3.1.1 Theoretical exposition

The privacy protection dilemma posed by BCI can be understood from two dimensions. First, the specificity of the privacy object. Neural signals directly collected by BCI not only reflect physiological states but also contain deep psychological information such as thought content, emotional tendencies, and cognitive preferences, thereby forming a “neural privacy” protection dilemma distinct from conventional data (32, 38). Second, the specificity of the data process. Unlike traditional medical data, neural data collection possesses both “passivity” and “continuity": passive BCI can capture brain activity signals generated involuntarily by users without their awareness (39), while continuous monitoring in long-term care settings means that privacy exposure risks permeate the entire process of collection, transmission, storage, and sharing. Moreover, EEG signals possess biometric identification properties, and once leaked, may trigger cascading risks such as identity theft and medical fraud (40). The above specificities give rise to a deep ethical tension: how to strike a reasonable balance between strengthening neural data protection and maintaining technological innovation vitality—this is the core focus of scholarly debate (41).

#### 3.1.2 Empirical evidence

Privacy and data security is the most frequently discussed ethical issue in the included literature, accounting for 28% of the total (32, 34, 38).

In the data collection phase, the study by Zander and Kothe (39) reveals that passive BCI can capture brain activity signals generated involuntarily by users, with most users unaware that their neural information is being recorded. In the transmission and storage phase, Cui et al. (12) point out that signals from wireless devices using protocols such as Bluetooth 5.0 face the risk of illegal interception, and existing encryption technologies cannot provide comprehensive protection for brain signals. In the data sharing phase, Nicolas-Alonso and Gomez-Gil (8) found that some institutions share neural data without obtaining complete informed consent or with insufficient anonymization, constituting clear compliance deficiencies. The survey by Yadav and Maini (42) shows that BCI industry practitioners generally regard neural privacy protection as the primary ethical challenge.

At the level of response strategies, scholarly positions diverge sharply: Ienca and Andorno (32) and others advocate for strengthening data encryption, tiered access management, and specialized “neural privacy” legislation; whereas Boonstra (41) cautions that excessive regulation may impede technological innovation and delay access for patient populations who stand to benefit most.

### 3.2 Informed consent and autonomous decision-making ethics

#### 3.2.1 Theoretical exposition

Informed consent constitutes the foundational ethical principle for the clinical application of BCI. However, in elderly care settings, elderly populations, patients with cognitive impairments, and severely disabled individuals face multiple implementation barriers due to cognitive decline and insufficient health literacy: first, BCI integrates multidisciplinary knowledge from neuroscience, engineering, and biomedical sciences, and the high complexity of technical principles makes it difficult for users to substantively understand surgical risks and potential hazards (43); second, some users may accept intervention under pressure from family members or healthcare providers, creating risks of “implicit coercion”; third, technology iterates rapidly and new risks continue to emerge during long-term use, yet initial consent cannot cover dynamic changes, creating institutional gaps in dynamic informed consent (44); fourth, for populations without autonomous decision-making capacity, existing surrogate decision-making mechanisms lack unified ethical norms and decision-making standards (45). Scholars generally call for the design of more adaptive and humanized informed consent processes for vulnerable populations.

#### 3.2.2 Empirical evidence

Informed consent and autonomy issues receive significant attention in 22% of the included literature (46).

Regarding information comprehension barriers, the clinical observations by Williams et al. (43) show that BCI involves interdisciplinary technical terminology, and even after simplification, users still struggle to accurately grasp key risks such as surgical complications of invasive devices and algorithmic decoding errors. King et al. (38) further point out that technical complexity prevents a large number of users from truly understanding the risk terms in consent forms. The study by Gosseries et al. (47) on patients with severe cognitive impairment and amyotrophic lateral sclerosis confirms that this population generally cannot independently complete the informed consent process.

Regarding institutional deficiencies, Sellers et al. (45) reveal the normative gap at the surrogate decision-making level—there is currently no unified ethical standard or decision-making process for populations without autonomous decision-making capacity. Wilkins et al. (44) further note that BCI technology iterates rapidly, and long-term use may give rise to new issues such as device compatibility changes, misjudgment risks from algorithm updates, and emerging data security vulnerabilities, yet the current system lacks institutional design for continuous consent updating. Regarding improvement pathways, Wilkins et al. (44) recommend using plain language and visual tools to simplify information presentation, establishing cooling-off periods, and creating dynamic consent update mechanisms.

### 3.3 Personhood and identity ethics

#### 3.3.1 Theoretical exposition

By establishing a direct information pathway between the human brain and machines, BCI may profoundly alter users' self-perception, behavioral patterns, and social relationships. Long-term use tends to trigger four types of issues: first, blurring of human-machine boundaries, where users internalize the device as part of their body schema, and device failure or replacement may precipitate identity crises (48); second, excessive reliance on device feedback functions, gradually leading to loss of independent decision-making and emotional regulation capabilities, forming technological dependence (26); third, invasive neural stimulation may affect emotional stability and social behavior, triggering personality trait changes (42); fourth, device-mediated communication patterns alter social interaction styles, causing interpersonal alienation and social identity dissolution (33). The core academic controversy in this field centers on the causal attribution of personality changes to technological intervention.

#### 3.3.2 Empirical evidence

Personhood and identity issues are discussed in 26% of the included literature (48).

Regarding human-machine boundary blurring, the qualitative study by Gilbert et al. (48) found that elderly and disabled users of long-term BCI tend to perceive the device as part of their body, and device failure or replacement triggers noticeable self-identity disturbances. Livanis et al. (26) observed that some users (including long-term elderly users) gradually cede daily decision-making and emotional regulation authority to the device, forming difficult-to-reverse patterns of technological dependence.

Regarding personality traits and social identity, Yadav and Maini (42) point out that the neural stimulation functions of invasive BCI (particularly deep brain stimulation combined with BCI) may affect users' emotional stability and social behavioral patterns. Sample et al. (33) found that device-mediated communication patterns alter the interaction rhythms between users and others, with some users consequently experiencing interpersonal alienation and repositioning of their social identity.

Regarding the attribution controversy, the systematic review by Klein and Ojemann (49) indicates that existing evidence is insufficient to attribute personality changes primarily to technological intervention—the effects of the disease itself, medication regimens, and living environments may be more significant. Some scholars therefore advocate for strengthening risk disclosure and long-term follow-up, while others contend that environmental and disease factors have greater impact.

### 3.4 Technical risks and use safety ethics

#### 3.4.1 Theoretical exposition

Elderly populations experience decline in physiological functions, wound healing capacity, and emergency response speed, and their tolerance for device failures and medical interventions is significantly lower than that of the general population, making safety issues of BCI in elderly care applications particularly prominent. Relevant risks can be categorized into four types: immediate physiological injury from invasive surgery (infection, bleeding, nerve damage, etc.) (50); nursing operation errors caused by decoding deviations during device operation (51); performance degradation and tissue reactions from long-term use of implants; and the absence of emergency switchover solutions in sudden failure scenarios (52). These risks directly threaten users' life, health, and care safety. Industry research focuses on hardware optimization, algorithm upgrades, and emergency system development (King et al., 2024).

#### 3.4.2 Empirical evidence

Literature focusing on technical risks and safety accounts for 16% of the included literature (36).

Regarding surgical and implantation risks, the retrospective analysis by Ramsey et al. (50) shows that the incidence of complications such as infection, bleeding, nerve damage, and cerebrospinal fluid leakage from invasive electrode implantation is significantly higher in elderly patients than in younger populations, with longer postoperative recovery periods.

Regarding device operation and long-term use risks, Grübler et al. (51) point out that elderly users, due to declining brain activity stability and limb tremor interference, experience significantly increased signal decoding error rates, potentially triggering care equipment incorrectly or mis-transmitting help-seeking information. The systematic review by Mak and Wolpaw (10) shows that long-term wearing of non-invasive electrodes can cause skin irritation and contact problems. The five-year follow-up data from Colachis et al. (53) on implanted intracortical microelectrode arrays show a mean time to device failure of approximately 330 days, a maximum continuous stable use time of 5 years, and a general declining trend in neural signal-to-noise ratio with implantation duration. The comparative experiment by Volosyak, Gembler, and Stawicki (54) further confirms that the information transfer rate of steady-state visual evoked potential (SSVEP)-based BCI used by elderly individuals is significantly lower than that of younger populations, with correspondingly higher error rates.

Regarding emergency safeguards, Ugur et al. (52) reported multiple cases of users who rely on BCI for basic life functions suffering harm due to sudden failures and the lack of rapid switchover solutions. Regarding improvement directions, King et al. (38) systematically synthesize scholarly consensus, covering optimization of implant materials, enhancement of decoding algorithm robustness, establishment of regular device testing standards, and improvement of emergency mechanisms.

### 3.5 Equity and accessibility ethics

#### 3.5.1 Theoretical exposition

The core concern of equity and accessibility ethics is distributive justice of BCI technology across different populations. There are currently four major access barriers: economic cost barriers—high device costs and insufficient insurance coverage (33, 38); technical operation barriers—rural and less-educated populations lack professional training resources (55, 56); device adaptation barriers—existing products are mostly designed for younger populations, lacking optimization for elderly physiological characteristics such as scalp laxity and hand tremors (36, 37); and geographic distribution barriers—the gap between developed and developing countries in technology dissemination and medical support is substantial (32, 56). These barriers may further widen health disparities among populations of different income, education, and geographic backgrounds. Scholarly debate centers on the balance between “inclusive promotion” and “technological research and development efficiency” (57, 58).

#### 3.5.2 Empirical evidence

Literature addressing equity and accessibility issues accounts for 10% of the included literature (59).

Regarding economic accessibility, the surveys by Burwell et al. (33) and King et al. (38) show that invasive BCI devices cost up to hundreds of thousands of dollars, and even non-invasive devices cost several thousand dollars or more. Combined with insufficient medical insurance coverage and widespread denial by commercial insurance, low-income populations are essentially unable to afford them.

Regarding geographic and technical accessibility, Hwang et al. (56) and Ienca and Andorno (32) point out the substantial gap between developed and developing countries in BCI technology dissemination and medical support. d'Ascoli et al. (55) further reveal that rural and less-educated populations lack necessary device operation training resources, forming a clear “digital divide.” The empirical survey by Zaman (60) validates the above trend: BCI usage rates are significantly positively correlated with users' income levels and degree of urbanization.

Regarding insufficient device adaptation, Baniqued et al. (36) and Yu et al. (37) point out that existing devices are mostly designed based on physiological parameters of young, healthy populations, without optimization for elderly characteristics such as scalp laxity, weakened brain activity, and hand tremors, resulting in a considerable proportion of target users being unable to achieve good user experience.

Regarding policy orientations, El-Osta et al. (58) advocate for improving accessibility through government subsidies, technology inclusion policies, and low-cost device development; while Cabrera and Weber (57) adopt a cautious stance, arguing that excessive pursuit of equity may weaken the momentum of technological innovation.

### 3.6 Risk-Benefit trade-off ethics

#### 3.6.1 Theoretical exposition

Risk-benefit trade-off constitutes the core decision-making basis throughout all ethical issues in BCI elderly care applications. However, this trade-off faces multiple special difficulties: users, healthcare providers, researchers, and policymakers have different perspectives on the assessment of technological benefits (44); elderly individuals and family members have divergent risk perceptions, which may trigger family decision-making conflicts (49); long-term rehabilitation benefits may require bearing short-term risks such as surgical risks, privacy breaches, and economic costs, while the limited lifespan of elderly populations makes assessment more complex (61); and some technologies (such as emotional support and cognitive enhancement types) have not yet been fully validated clinically but have already entered market application (41). These factors greatly increase the difficulty of ethical decision-making and call for the establishment of individualized assessment mechanisms (62).

#### 3.6.2 Empirical evidence

Literature discussing risk-benefit trade-off accounts for 28% of the included literature (63).

Regarding cognitive differences among assessment stakeholders, Wilkins et al. (44) found systematic differences in evaluation criteria among different stakeholders: users prioritize life autonomy, healthcare providers focus on care efficiency improvement, researchers concentrate on technological performance breakthroughs, and policymakers prioritize cost-effectiveness and population-level equity.

Regarding public cognition and family decision-making, the large-sample survey by Chen et al. (64) shows that over half of the general public holds overly high expectations that BCI can “fully restore motor/communication functions,” while actual clinical data show that the functional improvement rate for severely disabled users is approximately 40%. The survey by Grevet et al. (65) found that the majority of the public tends to believe that the overall benefits of BCI outweigh the risks, but the attitudes of patients' family members have a decisive influence on patients' willingness to use the technology. Klein and Ojemann (49) point out that elderly individuals tend to have lower vigilance toward technological risks, while family members often exhibit higher risk aversion—this cognitive gap easily triggers family decision-making conflicts.

Regarding therapeutic uncertainty, Boonstra (41) points out that emotional support and cognitive enhancement BCI lack high-quality randomized controlled trial evidence but have already entered market application and clinical trial stages. In response to the above dilemmas, Zhang et al. (62) systematically propose the establishment of an individualized risk-benefit assessment framework, emphasizing the need for comprehensive judgment incorporating users' health status, life needs, personal values, and economic capacity, while strengthening risk disclosure and benefit expectation management.

### 3.7 Interconnections among ethical themes

#### 3.7.1 Theoretical exposition

The six ethical themes above are not isolated from one another but form an interconnected network through multiple mechanisms. Based on literature synthesis, these interconnections operate primarily at three levels:

First, informed consent as a prerequisite safeguard, the degree of its implementation directly affects the realization of other ethical dimensions. When the informed consent process is not standardized, users' right to know and choose regarding the subsequent use of neural data cannot be guaranteed (44), and the risk of privacy breaches increases accordingly; simultaneously, users who lack adequate awareness of technological risks cannot make genuinely autonomous risk-benefit trade-offs.

Second, technical safety as the foundation of trust, the reliability and stability of devices affect users' overall trust in the BCI system, which in turn indirectly influences identity formation in the human-machine integration process. Frequent device failures and signal instability weaken users' psychological safety, hindering the process of internalizing the device as “part of the body” (48)

Third, accessibility as a structural condition, its deficiency not only directly constitutes distributive injustice but also excludes users from potential rehabilitation benefits and social participation due to inability to access the technology, forming a cumulative effect of ethical harm 69]. The rights of accessibility-limited populations—including informed consent, privacy protection, and risk-benefit assessment—remain effectively in suspension.

#### 3.7.2 Empirical evidence

Bibliometric analysis shows that technical risks and safety, privacy and data security, and informed consent and autonomy are the three most densely discussed ethical directions in the existing literature (33).

Regarding the association between informed consent and privacy protection, the study by Wilkins et al. (44)reveals the intrinsic connection between the two—when the informed consent process is not standardized, users' right to know and choose regarding the subsequent use of neural data cannot be guaranteed, and the risk of privacy breaches increases accordingly. The longitudinal study on human-machine integration by (48) further shows that frequent device failures and technical instability at the operational level significantly weaken users' psychological safety, thereby hindering the identity integration process of internalizing the device as “part of the body.”

Sawyer et al. (66), from a social justice perspective, point out that the “neural divide” caused by insufficient accessibility is not only a distributive injustice in itself but also excludes users from subsequent rehabilitation benefits and social participation, forming a compounding effect of ethical harm. Boonstra (41), at a macro level, warns that strategies that unilaterally emphasize potential benefits while neglecting risk prevention and control during technology dissemination can simultaneously trigger multiple ethical problems—privacy breaches, safety hazards, and trust collapse—creating a mutually reinforcing cycle among these issues.

## 4 Integrated analysis of ethical dilemmas and communication challenges

### 4.1 The transformation mechanism from ethical dilemmas to communication challenges

The six ethical dilemmas identified above are not limited to abstract ethical discussions—they inevitably transform into specific communication challenges in clinical elderly care practice. Understanding this transformation mechanism is the key to moving from ethical analysis to practical intervention.

From privacy dilemmas to communication challenges. The sensitivity of neural data requires not only technical encryption protection but also healthcare providers' ability to clearly explain to users “what data is being collected,” “where the data will flow,” and “what leakage risks may be faced.” However, when clinical staff lack systematic understanding of BCI data processes themselves (67), the institutional promises of privacy protection cannot be translated into users' informed choices through effective communication.

From informed consent dilemmas to communication challenges. The core obstacles to informed consent—information comprehension difficulties, implicit coercion, and lack of dynamic consent—are essentially ethical expressions of communication failure. When the cognitive gaps described earlier exist between doctors and patients, no formatted consent form can bridge the comprehension gap. This requires communication strategies to shift from “one-time notification” to “continuous dialogue.”

From risk-benefit trade-off dilemmas to communication challenges. The divergent perceptions of risks and benefits among different stakeholders (44) necessitate the establishment of multi-directional communication mechanisms, enabling users' lived experiences, family members' concerns, clinical professionals' judgments, and researchers' technical explanations to intersect on a shared deliberative platform.

### 4.2 Three-Layer communication challenge framework

Based on the above transformation logic, and combined with empirical reports on communication barriers in the included literature, the communication challenges in BCI elderly care applications can be summarized into three progressive layers:

- Layer 1: Human-Computer Interaction Level Communication Challenges. This layer refers to information exchange barriers between users and BCI devices, directly stemming from the physical and engineering limitations of the technical system as well as mismatches with users' physiological and cognitive characteristics. Technical-side difficulties include signal noise interference (68, 69), device performance degradation (53, 82), wireless transmission errors (70), and signal quality loss due to power-performance trade-offs (71). User-side difficulties include extended training periods due to age-related decline in neural regulatory capacity (72), decreased control accuracy (73), and the impact of individual alpha frequency differences on interaction efficiency (35). The superimposition of technical-side and user-side difficulties means that elderly user populations face far higher interaction thresholds than the general population, directly affecting device accessibility and user experience.
- Layer 2: Interpersonal Communication Level Communication Challenges. This layer refers to communication barriers among multiple stakeholders—users, family members, healthcare providers, and researchers—arising from professional knowledge gaps, expectation differences, and interdisciplinary collaboration breakdowns. Empirical studies show that clinical healthcare providers generally lack systematic BCI training and cannot provide patients and families with accessible and accurate technical explanations and risk disclosures (67); family members have excessively high expectations for rehabilitation outcomes, conflicting with patients' actual experiences (74); and engineers and clinical professionals have inconsistent evaluation criteria, leading to decoupling in interdisciplinary collaboration (74, 75). Content attenuation and distortion in multi-level information transmission further amplify communication difficulties (76).
- Layer 3: Social Cognition Level Communication Challenges. This layer refers to the cognitive gap between media and the public caused by information distortion and the absence of science communication. Media reporting generally exhibits an optimistic bias that exaggerates therapeutic efficacy, with over 70% of BCI reports downplaying technical risks (77) and shaping public cognition through misleading concepts such as “mind control” and “mind reading” (6). False or exaggerated content spreads approximately 70% faster on social media than professional science communication (78). Globally, BCI reporting is concentrated in Western and East Asian media, with localized discussions and risk assessments in emerging economies largely absent (77), and the lack of professional science communication channels prevents authoritative knowledge from reaching the general public (79). These factors collectively constitute systematic communication barriers at the social cognition level.

### 4.3 Cross-Level integration mechanism of human-machine trust

“Human-machine trust” serves as a cross-level integrating mechanism within this study's theoretical framework, and its formation and maintenance depend on the synergistic operation of the three communication levels described above.

Conceptually, human-machine trust can be defined as a psychological state in which users, in an uncertain technological environment, believe that the BCI system can reliably and safely achieve its intended functions while safeguarding user interests. Unlike human-machine trust in general contexts, trust in BCI scenarios has two distinctive features: first, the object of trust includes not only hardware devices and software algorithms but also the collection and decoding of neural signals—a technical link that directly affects the user's “inner self"—making the psychological involvement in trust far exceed that of ordinary technological products (48); second, the establishment of trust depends not only on individual user experience but is also significantly moderated by social factors such as family attitudes, media coverage, and clinical staff recommendations (65, 74), exhibiting “social embeddedness” characteristics.

Operationally, the formation of human-machine trust depends on communication effects at three levels: signal stability and feedback accuracy at the human-computer interaction level (53) constitute the technical foundation of trust; information transparency and expectation management at the interpersonal communication level (44) constitute the social foundation of trust; and science communication and cognitive correction at the social cognition level (6, 77) constitute the public opinion foundation of trust. All three are indispensable: frequent technical failures erode users' perceptions of device reliability (48); poor doctor-patient communication leads to magnification or underestimation of technological risks (49); and media misinformation generates expectations or fears at the public level that are disconnected from the technology's actual performance (6). Failure at any single level can undermine overall trust, leading to user withdrawal from use, family of decisions, or public resistance to technology adoption.

From a dynamic perspective, human-machine trust has three developmental stages: formation, consolidation, and decline/repair. Trust formation – from first contact to initial use – hinges critically on information transparency and risk communication is particularly critical (44). Its consolidation during long-term use depends jointly on sustained device reliability and consistent operational support from healthcare providers (48). When trust declines following device failure or adverse events, its restoration turns on the clarity of emergency communication mechanisms and the transparency of responsibility attribution (52). This dynamic perspective suggests that trust management should not be a one-time publicity or notification effort, but should run through the entire life cycle of technology application.

## 5 Research limitations

This section systematically examines the limitations of this study from both methodological and epistemological perspectives.

### 5.1 Methodological limitations

#### 5.1.1 Absence of quality assessment of included literature

This review completed literature retrieval and screening following PRISMA guidelines but did not undertake systematic methodological quality assessment of the included literature (e.g., using QUADAS-2, CASP, or mixed-methods evaluation tools). This implies that the empirical evidence synthesized in this review may suffer from variations in quality across original study designs, sample representativeness, data collection, and analytical methods. Although we prioritized citing literature with clearly defined research designs and sample sources when summarizing the “empirical evidence” for each theme (e.g., the qualitative study by Gilbert et al. (48), the five-year follow-up data from Colachis et al. (53), the large-sample survey by Chen et al. (6), we did not systematically assign quality scores or provide stratified reporting of the quality levels of the included literature. This gap may affect the reliability and persuasiveness of the evidence synthesis in this review.

#### 5.1.2 Heterogeneity of evidence sources and integration challenges

The included literature exhibits significant heterogeneity in research design, target populations, technology types, and outcome measures. Specifically: some studies employ qualitative interview designs (e.g.,Gilbert et al.; Wilkins et al.) (44, 48), focusing on exploring users' subjective experiences and attitudes; some adopt quantitative surveys (e.g., Grevet et al., Zaman) (60, 65), emphasizing the reporting of frequency distributions and correlations; others utilize technical experimental designs (e.g., Colachis et al.; Volosyak et al.) (53, 54), focusing on device performance metrics; and a considerable portion consists of conceptual ethical analyses (e.g., Ienca & Andorno; Boonstra,) (32, 41). These different types of evidence differ fundamentally in methodological paradigms, strength of evidence, and generalizability. Incorporating them into the same review framework for comprehensive synthesis entails certain risks of integration heterogeneity. Although we distinguished the functions of different types of literature through the dual structure of “theoretical exposition” and “empirical evidence,” we did not assign differentiated weights or confidence ratings to evidence derived from different study designs.

#### 5.1.3 Publication bias and positive results bias

As a rapidly developing emerging field, brain-computer interface research may be subject to typical publication bias—studies reporting positive results, significant effects, or technological breakthroughs are more likely to be published, while studies reporting negative outcomes, device failures, or ethical difficulties may be underestimated due to journal preferences. In this review, empirical studies on “technical risks and use safety” (e.g., device failures, complications, lack of emergency responses) are relatively scarce, accounting for only 16% of the included literature (36). Considering that the actual frequency of technical failures and safety incidents in elderly care practice may be higher than this proportion, the current literature distribution may underestimate the true severity of technical safety issues. Furthermore, this review excluded non-English language literature and grey literature (e.g., technical reports, conference abstracts, industry white papers), which may further exacerbate the impact of publication bias—such literature often contains more information about technical failures, implementation difficulties, and unanticipated risks.

### 5.2 Epistemological limitations

#### 5.2.1 Geographic concentration of evidence and cultural applicability

The vast majority of literature included in this review originates from high-income countries (particularly the United States, EU member states, and developed East Asian economies), which is consistent with Beck et al.'s (77) finding that BCI reporting is concentrated in Western and East Asian media. This geographic concentration entails two limitations:

First, research findings from high-income countries may not be applicable to nursing contexts in low- or middle-income countries. The latter differ structurally from high-income countries in terms of infrastructure (e.g., stable electricity supply, network coverage, healthcare resources), nursing human resource allocation, and cultural values (e.g., family caregiving models, trust in technological authority)—differences that may fundamentally alter the manifestations and priorities of ethical issues.

Second, even the same ethical theme (e.g., “privacy protection” or “informed consent”) may carry entirely different practical implications across cultural contexts. For instance, the expression of autonomous decision-making in collectivist cultural contexts differs significantly from that in individualist cultural contexts, yet the literature upon which this review relies has not provided differentiated examinations of this cultural dimension.

In Section 3.5 “Equity and Accessibility Ethics,” we have noted that “the gap in BCI technology popularization between developing and developed countries is large” (32, 56) and reported the existence of geographic accessibility barriers. However, the analytical framework of this review itself remains predominantly based on literature from high-income countries—this epistemological limitation implies that the six-theme ethical framework proposed herein may require cultural adaptation and priority reordering when applied to different cultural-economic contexts.

#### 5.2.2 Subjectivity of thematic classification

This review summarizes the ethical challenges into six themes—privacy protection, informed consent, personhood and identity, technical safety, equity and accessibility, and risk-benefit trade-off—a classification scheme derived from systematic reading and thematic coding of the included literature. Although we have reflected the literature base of the classification by reporting the proportion of literature addressing each theme (ranging from 10% to 28%), it must be acknowledged that any qualitative research's thematic classification process involves the researchers' subjective judgment and academic preferences. Different research teams might adopt different classification logics—for example, subsuming “personhood and identity” under a broader “autonomy” category, or splitting “technical safety” into two independent themes of “physical safety” and “information security.” Moreover, there are evident conceptual overlaps and boundary ambiguities among the six themes (e.g., privacy breaches are both a safety issue and an infringement of autonomy; technical risks directly affect risk-benefit trade-offs), making it difficult to achieve complete mutual exclusivity and exhaustiveness in practical thematic classification. While we have acknowledged the interconnections among themes in Section 3.7 “Interconnections among Ethical Themes,” this supplementation does not entirely eliminate the subjective influence inherent in the classification itself.

### 5.3 The anticipatory nature of ethical concerns

#### 5.3.1 Anticipatory Characteristics of Ethical Concerns and Uncertainty of Empirical Basis

A considerable portion of the ethical concerns discussed in this review—particularly those involving “neural privacy,” “personality changes,” “cognitive enhancement,” and “social identity alienation"—exhibits significant anticipatory or forward-looking characteristics. Specifically:

On the one hand, some ethical risks have not yet been fully realized or widely manifested at the current stage of BCI technology development. For example, concerns about “inadvertent decoding of inner speech” (80, 81) remain at the laboratory exploration stage and have not yet become a widespread practical threat in elderly care settings; the risk of “personality trait changes due to long-term use” is currently based primarily on observations of patients with deep brain stimulation (42), and its extension to BCI users remains within the realm of theoretical inference; applications of “cognitive enhancement” BCIs in elderly care are still in early experimental stages (41).

On the other hand, the basis for discussing these anticipatory ethical concerns largely consists of “prospective analyses” or “precautionary ethical analyses” based on current technological trends, rather than “retrospective empirical summaries” grounded in widespread clinical practice. This means that a considerable portion of the evidence synthesized in this review belongs to “predictive evidence” rather than “verified evidence"—the former reflects experts' judgments about future possibilities, while the latter reflects clinical facts that have already occurred.

This anticipatory nature imposes important constraints on the nature of this review's conclusions: the ethical challenges described herein are partly observable practical issues in clinical settings (e.g., privacy breaches, difficulties with informed consent, surgical complications), but partly remain theoretical inferences based on technological development trends (e.g., neurorights protection, cognitive enhancement and transhumanist ethics). Although the two are formally distinguished through the dual structure of “theoretical exposition” and “empirical evidence,” readers should still be aware when interpreting that content presented under “empirical evidence” does not all enjoy the same “verified” status—some are preliminary observations based on limited clinical data, while others are expert opinions or prospective analyses.

Therefore, this review is essentially a construction of a predictive ethical analysis framework, rather than an empirical investigation report on “already occurred ethical issues.” We recommend that when applying this framework to practice, readers distinguish between those ethical concerns that already have a clear empirical basis (e.g., infection risks of invasive devices, cognitive barriers to informed consent) and those that remain at the stage of theoretical inference (e.g., the right to personality continuity, the ethical boundaries of cognitive enhancement), and continuously track how technological developments verify or revise the actuality of ethical risks.

### 5.4 Strengths and boundaries of this review

Notwithstanding the aforementioned limitations, this review, through its systematic PRISMA-based literature retrieval and screening process, clear inclusion and exclusion criteria, stepwise refinement and multiple validation of thematic classification, and the dual presentation structure of “theoretical exposition” and “empirical evidence,” provides operational conceptual tools and an analytical framework for ethical analysis of brain-computer interfaces in elderly care settings. Future research may build upon this review by incorporating quality assessment tools (e.g., QUADAS-2, CASP), expanding sources to include non-English language literature and grey literature, conducting cross-cultural comparative analyses, and tracking empirical validation of the actuality of ethical concerns as technology develops, so as to continuously improve the evidence base and analytical framework in this field.

## 6 Conclusion

This systematic review has mapped the ethical landscape of BCI research across 177 studies, identifying six overarching challenge domains and demonstrating that these challenges are not abstract philosophical concerns but are structurally embedded in the interaction chain of neural signal acquisition, intention decoding, interface presentation, and care execution. Three cross-cutting findings warrant particular emphasis. First, the probabilistic nature of neural decoding is not an engineering limitation to be eventually resolved; it is an inherent epistemic condition that care workflows must accommodate through explicit uncertainty communication, secondary confirmation protocols, and fail-safe mechanisms for safety-critical actions. Second, neural data privacy cannot be adequately addressed by data protection frameworks designed for conventional health records: as decoding algorithms advance, the inferential boundary of BCI data expands beyond its original intended scope, requiring a category-specific governance response. Third, trust in BCI-mediated care is not a product of user education or device accuracy alone, but a relational outcome that depends on explainable outputs, traceable decision pathways, and the preservation of caregiver-patient communication-all of which must be designed into systems from the outset rather than retrofitted.

These findings translate into four specific recommendations for researchers, clinicians, and policymakers. (1) Regulatory agencies should establish a BCI-specific neural data category within existing health data law, with protections covering not only raw signal storage but downstream inferences, and should mandate that device manufacturers disclose the full scope of psychological states their systems are technically capable of inferring at the point of market authorisation. (2) Clinical trial ethics review must mandate a staged, capacity-adapted informed consent process for BCI studies involving older adults or cognitively impaired participants, one that includes an initial simplified information session, a mandatory reflection period, and documented reassessment of consent at each major protocol change. (3) Healthcare institutions deploying BCI should operationalise a minimum viable ethics implementation standard comprising three operationally defined features: a real-time confidence indicator visible to both user and caregiver, a user-accessible pause-and-exit mechanism operable without full BCI control, and an auditable log of all decoded commands accessible to the care team for review. (4) Funding bodies should prioritise research that recruits older adults as design partners in BCI development rather than solely as end-stage trial participants, and should require equity impact assessments addressing age-related signal variability and socioeconomic accessibility barriers as conditions of grant approval for translational BCI projects.

The translation of BCI from laboratory capability to responsible elderly care practice is ultimately not a problem of technological maturity alone. It is a problem of institutional readiness: whether the governance, clinical, and design infrastructures surrounding these systems are sufficiently robust to honour the ethical commitments that their deployment implicitly entails. This review has sought to provide a structured foundation for that work.

## Data Availability

The raw data supporting the conclusions of this article will be made available by the authors, without undue reservation.
